# Integration Analysis of Small RNA and Degradome Sequencing Reveals MicroRNAs Responsive to *Dickeya zeae* in Resistant Rice

**DOI:** 10.3390/ijms20010222

**Published:** 2019-01-08

**Authors:** Wenqi Li, Yulin Jia, Fengquan Liu, Fangquan Wang, Fangjun Fan, Jun Wang, Jinyan Zhu, Yang Xu, Weigong Zhong, Jie Yang

**Affiliations:** 1 Institute of Food Crops, Jiangsu Academy of Agricultural Sciences/Nanjing Branch of Chinese National Center for Rice Improvement, Nanjing 210014, China; liwenqi@jaas.ac.cn (W.L.); qiezi324@163.com (F.W.); weidadefan@sohu.com (F.F.); wangjunjaas@aliyun.com (J.W.); zhujinyanrain@163.com (J.Z.); linkinxy@163.com (Y.X.); wgzhong0503@139.com (W.Z.); 2 Jiangsu Co-Innovation Center for Modern Production Technology of Grain Crops, Yangzhou University, Yangzhou 225009, China; 3 United States Department of Agriculture-Agriculture Research Service, Dale Bumpers National Rice Research Center, Stuttgart, AR 72160, USA; yulin.jia@ars.usda.gov; 4 Institute of Plant Protection, Jiangsu Academy of Agricultural Sciences, Nanjing 210014, China; fqliu20011@sina.com

**Keywords:** rice foot rot disease, *Dickeya zeae*, microRNA, RNA and degradome sequencing, disease resistance

## Abstract

Rice foot rot disease caused by the pathogen *Dickeya zeae* (formerly known as *Erwinia chrysanthemi* pv. *zeae*), is a newly emerging damaging bacterial disease in China and the southeast of Asia, resulting in the loss of yield and grain quality. However, the genetic resistance mechanisms mediated by miRNAs to *D*. *zeae* are unclear in rice. In the present study, 652 miRNAs including osa-miR396f predicted to be involved in multiple defense responses to *D. zeae* were identified with RNA sequencing. A total of 79 differentially expressed miRNAs were detected under the criterion of normalized reads ≥10, including 51 known and 28 novel miRNAs. Degradome sequencing identified 799 targets predicted to be cleaved by 168 identified miRNAs. Among them, 29 differentially expressed miRNA and target pairs including miRNA396f-*OsGRFs* were identified by co-expression analysis. Overexpression of the osa-miR396f precursor in a susceptible rice variety showed enhanced resistance to *D. zeae*, coupled with significant accumulation of transcripts of osa-miR396f and reduction of its target the *Growth*-*Regulating Factors* (*OsGRFs*). Taken together, these findings suggest that miRNA and targets including miR396f-*OsGRFs* have a role in resisting the infections by bacteria *D. zeae*.

## 1. Introduction

Plant microRNAs (miRNAs) are non-protein coding RNAs with 21–24 nucleotides (nt). Most recently, miRNAs were demonstrated to be a critical component of sophisticated plant defense system [1,2,3]. Plants are known to use a multifaceted defense system to prevent infections by pathogenic microbes through a co-evolutionary mechanism with pathogens. Plant immunity is involved in two tiers of defenses where the first tier is a pathogen-associated molecular pattern (PAMP) trigged immunity (PTI) [4,5]. The second tier of defense is often involved in pathogen effector-triggered immunity (ETI) mediated mostly by R proteins with leucine rich repeat and nucleotide binding site (NLR) [4,5]. Activation of ETI often reinforces PTI to achieve plant immunity to prevent damages by invaders [6,7,8]. It was predicted that targets of plant miRNAs can be NLR genes, or genes involved in PTI [2,3]. To name a few, Li et al showed that two miRNA, nta-miR6019 and nta-miR6020 co-regulated the transcripts of *R* genes to regulate resistance responses and productivity in tobacco [9]. In *Arabidopsis*, bacterial flagellin flg22-induced miR393 promoted resistance response to virulent *Pseudomonas syringae* pv. *tomato Pto* DC3000 by inhibiting predicted target-auxin receptor transcripts [10]. Three bacterial flagellin flg22-induced miRNAs, miR160, miR398b and miR773 were shown to be involved in resistance responses via regulating PAMP-induced callose deposition in *Arabidopsis* [11,12]. The stress-inducible miR163 and its target histone deacetylase were demonstrated to confer resistance to *P. s.* pv. *Tomato* [13].

In rice, recent studies also showed that miRNAs were involved in disease resistance [14,15,16]. Examples are 23 miRNAs and 59 targets were predicted to be involved in resistance against rice sheath blight disease [17]. A group of miRNAs and their targets functioned in an incompatible interaction between rice and fungal pathogen *Magnaporthe oryzae* (*M. oryzae*) [18]. In addition, the large differentially expressed miRNAs and their putative target genes were involved in hormone biosynthesis and signaling pathways in rice after infections by bacterial pathogens [19,20]. The osa-miR169, miR160a, miR398b, miR7695 and miR1861k repressed the expression of the predicted targets respectively, have a role in resistance to *M. oryzae* or *Xanthomonas oryzae* pv. *oryzae* (*Xoo*) [18,21,22,23]. 

Rice foot rot disease caused by the pathogenic bacteria *D. zeae*, is a newly emerged destructive bacterial disease threatening rice yield and quality [24]. The occurrence of rice foot rot disease was first reported in Japan in 70 s of the 20th century [25]. Recently, field incidence of rice foot rot disease shows an increasing trend in rice planting areas in Southeast Asia and South China [24]. Little is known about the pathogenicity of *D. zeae* and the genetic resistance mechanism to *D. zeae* [26,27,28,29], and it is unknown if miRNAs are involved in the defense responses to *D. zeae* in rice. Uncovering the resistance mechanism will lead to developing better control methods to this disease.

We previously isolated and identified an isolate of *D. zeae* JS2012 from diseased roots of a rice variety [30]. The objectives of the present study were to (1) determine temporal transcriptome profiles of miRNAs after *D. zeae* infection, (2) predict miRNA targets by degradome sequencing, (3) validate differentially expressed miRNAs and targets by qRT-PCR, and (4) overexpress the osa-miR396f precursor to determine if enhanced resistance to *D. zeae* occurs in rice. Through these efforts we demonstrated that a set of candidate miRNAs and relevant targets including miR396 and predicted target may play an important role in the defense responses to *D. zeae.*

## 2. Results

### 2.1. Overview of miRNA Sequencing 

A total of 4 miRNA libraries were constructed from a resistant rice variety (an incompatible interaction) at 0 h (K0), 6 h (K1), 12 h (K2) and 48 h (K3) after *D. zeae* inoculation. The illumina sequencings revealed 1,053,550 (K0), 1,000,514 (K1), 1,023,748 (K2), 483,076 (K3) unique clean reads respectively after removing low resolution, length less than 18 nt or greater than 25 nt, junk reads, mRNA, rRNA, tRNA, snRNA and snoRNA (Table 1). A total number of 19–24 nt from K0 miRNA library accounted for 82.82%, in which 21, 24 nt unique miRNA accounted for 16.10% and 11.03% respectively (Table S1). After inoculation with *D. zeae* in a resistant rice variety, the length of unique miRNAs was found between the range of 20–24 nt. Among them, 21 and 24 nt unique miRNAs accounted for 15.25% and 18.61% (K1), 17.15% and 14.83% (K2), 16.13% and 9.64%, respectively (K3; Table S1). Differences in miRNA length and number from different time points after pathogen inoculation may reflect different biological roles during different stages of rice growth and development.

### 2.2. Identification of Known and Novel miRNAs

**Figure 1:**
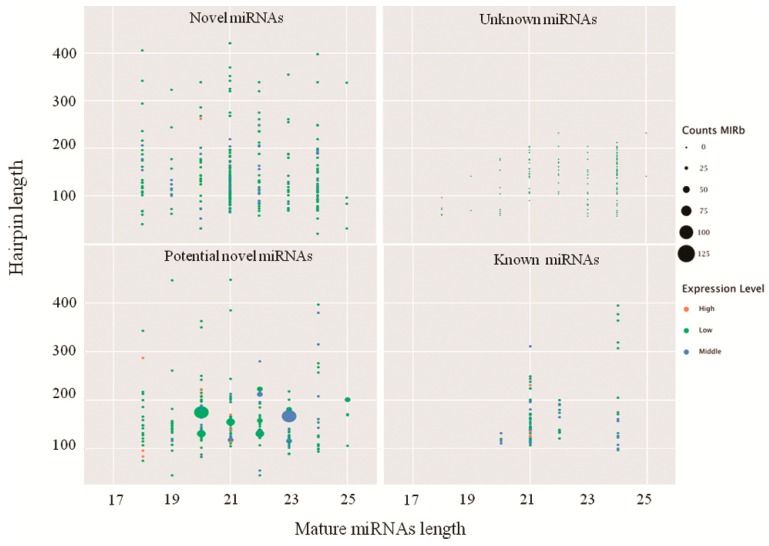
The four indicated groups of miRNAs identified from an incompatible interaction. CountsMIRb, the counts of miRNAs from miRBase. Expression level, low indicates <10, middle indicates >10 but less than average, high indicates over average.

A total of 652 miRNAs including 250 novel, 78 known, and 185 predicted but not mapped at the plant genome (potential novel miRNAs), and 139 mapped on the plant genome not in the miRNA database were identified (unknown miRNAs; Figure 1; Table S2). A total of 10 known miRNAs, osa-miR1425-5p, osa-miR156b-3p, osa-miR156c-3p, osa-miR171b, osa-miR156a, osa-miR535-5p, osa-miR162a, osa-miR166a-5p, osa-miR166a-3p and osa-miR166b-5p with more than 100 reads was detected (Figure 1; Table S2). Among them, three miRNA families, osa-miR156, 166 and 167, with the highest frequencies were identified (Figure 1; Table S2). The miRNAs mapped to the rice genome but did not match to known pre-miRNAs as novel miRNAs. 250 novel miRNAs were predicted in four libraries (Figure 1; Table S2). The majority of novel miRNAs was found to be slowly accumulated after inoculation with *D. zeae*. However, osa-MIR5083-p5 was the most abundant one with reads of 135.04 (K0), 484.00 (K1), 713.11 (K2) and 233.71 (K3) in four libraries, respectively, suggesting its unique role for responding to *D. zeae* infection in a resistant rice variety.

### 2.3. D. zeae-Responsive miRNAs in Resistant Rice

**Figure 2:**
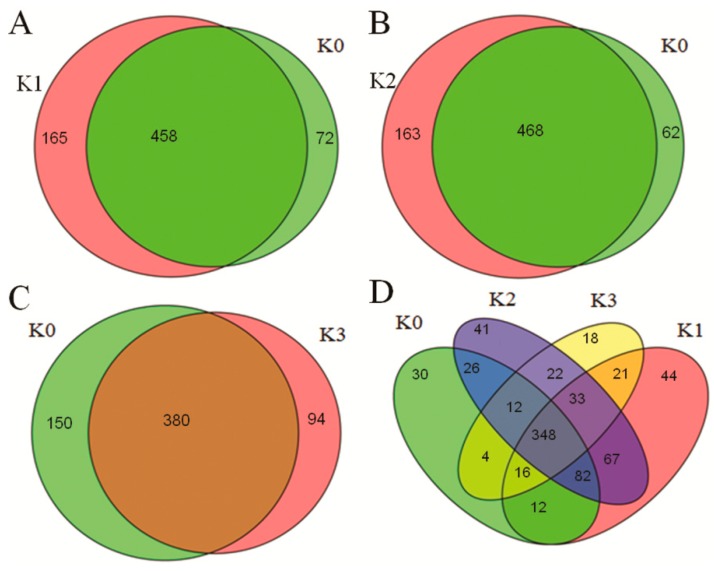
A summary of unique miRNAs detected in four libraries. (**A**–**C**) The miRNAs responded specifically the *D. zeae* infection in an incompatible interaction at 6, 12 and 48 h compared with at 0 h respectively. (**D**) The specific miRNAs were detected among four libraries.

To identify miRNAs in resistant rice that respond to *D. zeae*, we analyzed the differential expressions of miRNAs in the four libraries with the normalized reads from deep sequencing. A total of 79 differentially expressed miRNAs was found in all four libraries including 51 known miRNAs and 28 novel miRNAs under the criterion of normalized reads ≥10 (Figure 2A; Table S3). Furthermore, 34 miRNAs were up-regulated and 32 miRNAs were down-regulated at six hours post inoculation (hpi). At 12 hpi, 36 miRNAs were up-regulated and 32 miRNAs were down-regulated. At 48 hpi, 26 miRNAs were up-regulated and 39 miRNAs were down-regulated (Figure 2B; Table S3). These results suggest that certain miRNAs were expressed at some time points after *D. zeae* infection in an incompatible interaction suggesting that they may play an important role during pathogen infection. 

**Figure 3:**
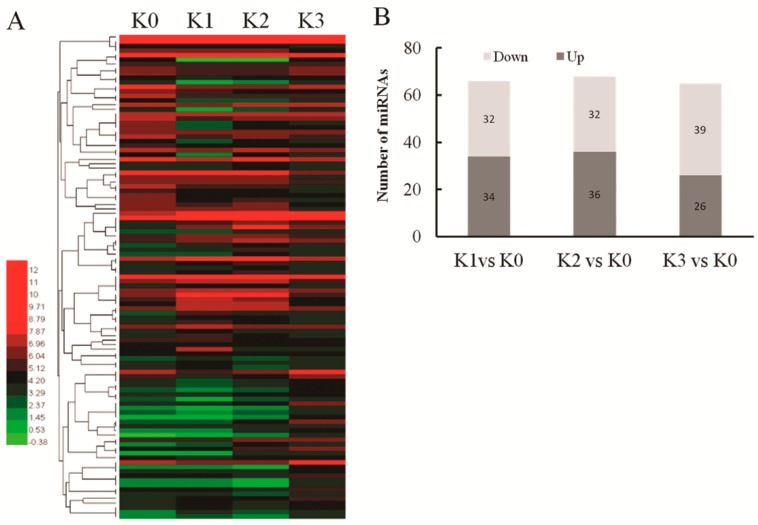
*D. zeae*-responsive miRNAs in resistant rice. (**A**) Heat plot showing *D. zeae*-responsive miRNAs identified in an incompatible interaction. (**B**) The number of differentially expressed miRNAs in four groups.

Sequence analysis revealed that there were 530 miRNAs in K0, 623 in K1, 631 in K2, and 474 in K3, respectively (Figure 3). When compared with K0, 165 unique miRNAs were identified at 6 hpi, (Figure 3A), 163 unique miRNAs were identified at 12 hpi, 94 unique miRNAs were detected at 48 hpi, respectively (Figure 3B,C). When compared among four libraries, 30 (K0), 44 (K1), 41 (K2) and 18 (K3) miRNAs were detected (Figure 3D). The presences of different miRNAs at different time points after inoculation suggest miRNAs have a role to *D. zeae* in an incompatible interaction.

### 2.4. Target Prediction of miRNAs by Degradome Sequencing and Analysis

To identify the relevant targets of miRNAs, high-throughput degradome sequencing was used and the data were analyzed with the CleavLand 3 pipeline. Degradome sequencing analysis showed that 10931903 and 8679632 raw reads were detected from the control (T0 h) and infected (T6–48 h) rice samples, respectively (Table S4). After removing the adaptor reads and mapping the rice transcriptome, 35890 and 32146 covered transcripts were identified in the control (T0 h) and infected (T6–48) rice samples, respectively (Table S4). Further, a total of 799 targets were predicted to be cleaved by the 168 identified miRNAs in an incompatible interaction (Table S5). Many transcript targets mediated by miRNAs were closely related to the signal transduction involved in plant immunity, such as PR5 (LOC_Os03g14030.1), leucine-rich repeat (LRR) kinase (LOC_Os12g10740.1), calcium-dependent kinase (LOC_Os07g22640.), WRKY transcription factor (LOC_Os04g51560.1), AUX/IAA transcriptional regulator (LOC_Os12g40900.1), ethylene-responsive binding protein (LOC_Os07g42510.1), growth-regulating factor 5 (LOC_Os02g53690.1) and etc (Table 2). These findings demonstrate that miRNAs-target pairs may be involved in resistance responses through multiple defense pathways in rice.

### 2.5. Functional Classification of Predicted Targets

**Figure 4:**
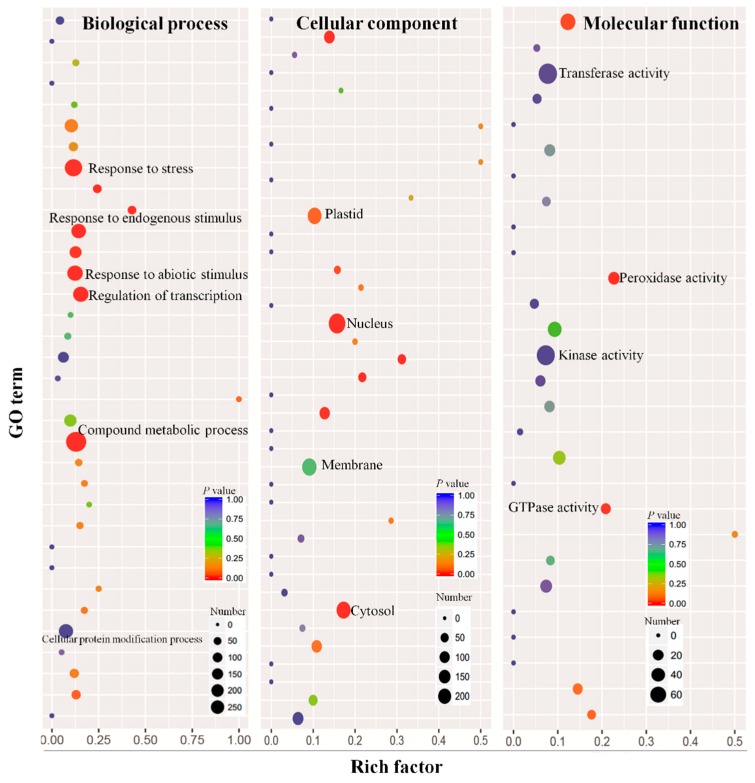
GO functional classification of identified target genes.

A gene ontology (GO) analysis was used to classify the biochemical functions of predicted targets (Figure 4). A total of 858 transcript targets were predicted as protein kinase, 852 as transferase, 171 as signal transducers, and 81 as protein receptors, suggesting their potential involvements in signal recognition and transduction in plant immunity (Figure 4). Similarly, a group of targets was predicted to be involved in multiple molecular function and all of them were predicted to be located in diverse cellular components of cells, suggesting that predicted target genes may also play a role in biological processes including hormone stimulus, signal transducer, sequence-specific DNA binding, secondary metabolic process, cell growth, and multicellular organismal development (Figure 4).

### 2.6. Co-expression Analysis and Validation of miRNA-Targets by qRT-PCR

**Figure 5:**
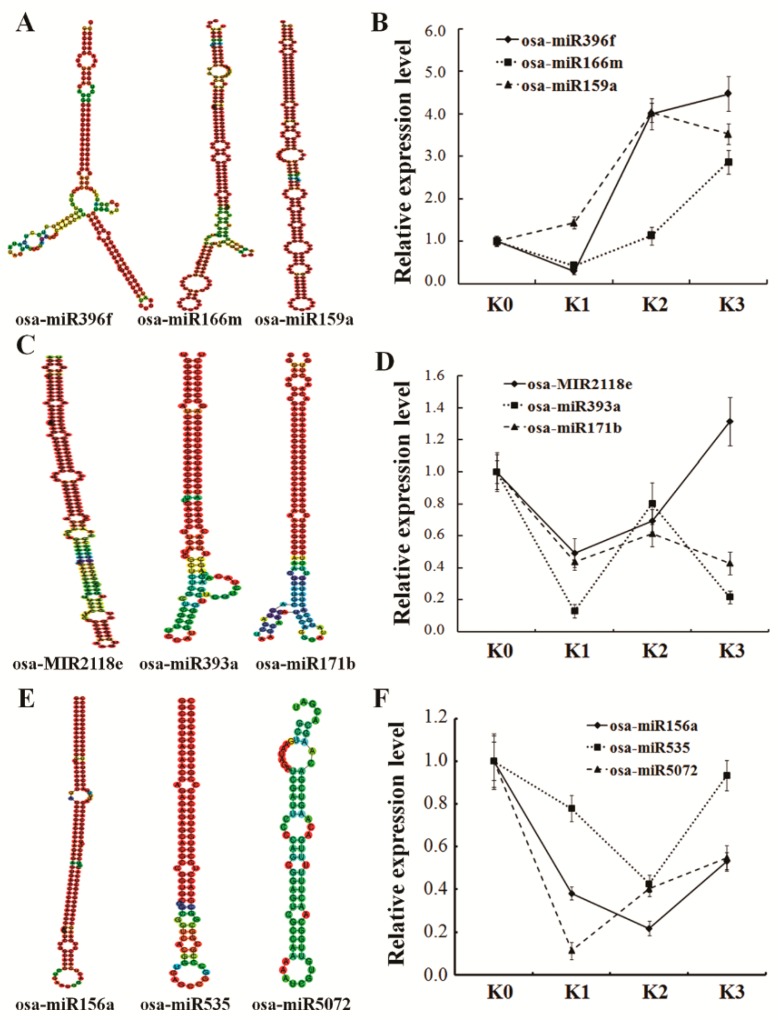
Predicted secondary structures and verification of expression of miRNAs precursor. (**A**,**C**,**E**) Predicted secondary structures of nine miRNAs precursor by RNAfold (http://rna.tbi.univie.ac.at//cgi-bin/RNAWebSuite/RNAfold.cgi, 19 March 2018) respectively. (**B**,**D**,**F**) The expression of nine miRNAs precursor were tested by qRT-PCR. K0, K1, K2 and K3 represent a resistance rice variety at 0, 6, 12 and 48 hpi respectively. Data represent means of three replicates ± standard deviation for each miRNA in the four miRNAs libraries.

**Figure 6:**
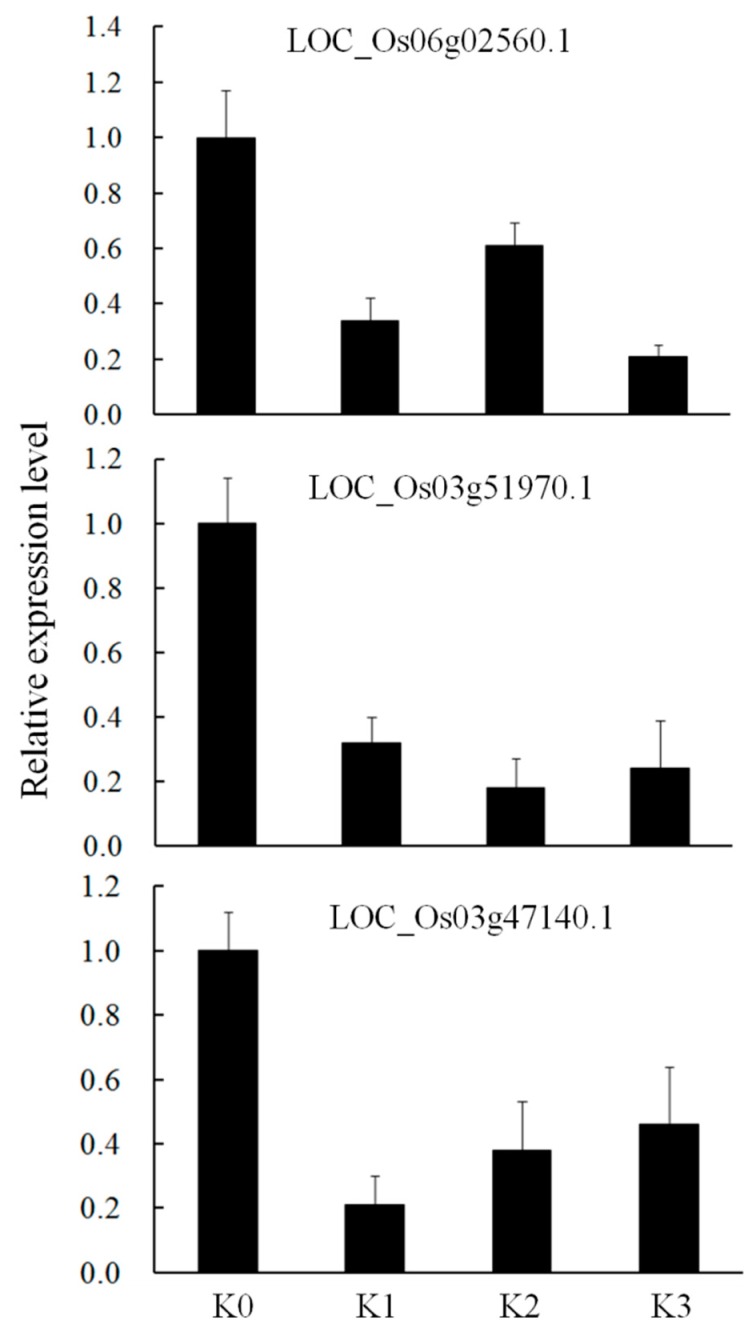
The expression levels of three targets *OsGRFs* of osa-miR396f analyzed by qRT-PCR in an incompatible interaction. Data represent means of three replicates ± standard deviation for each target in the four miRNAs libraries from an incompatible interaction.

We analyzed the miRNAs obtained by miRNA sequencing, and verified the data with qRT-PCR and degradome sequencing. Firstly, 29 (36.71%) of 79 differentially expressed miRNAs detected by miRNA-seq were detected from the degradome sequencing (Table S6). A total of 82 genes resulting in 132 transcripts were predicted to be targets of these 29 miRNAs (Table S6). Moreover, 9 differentially expressed miRNAs based on the number of normalized reads were selected from 29 miRNAs (Table 3). Interestingly, we found that among 9 miRNAs, 3 miRNAs were induced and 6 were reduced (Table S2). Moreover, the secondary structures of 9 miRNAs precursor were predicted by RNAfold respectively (Figure 5A). Despite the difference in the fold changes, the expression patterns of 9 miRNAs analyzed by qRT-PCR were consistent with that of miRNA-seq (Figure 5B). Moreover, the degradome sequencing data revealed an opposite expression pattern of the cognate targets of these 9 miRNAs (Tables S2 and S6) suggesting that these miRNAs may regulate target gene expression at different time points post inoculation in an incompatible interaction. Reduced expressions of three target genes, LOC_Os06g02560.1, LOC_Os03g51970.1 and LOC_Os03g47140.1) predicted to be regulated by osa-miR396f were verified by qRT-PCR, suggesting that the osa-miR396f-targets pair may play a role for resistance to *D. zeae* in rice (Figure 6).

### 2.7. Overexpression of Osa-miR396f Precursor Enhanced Rice Resistance to D. zeae

**Figure 7:**
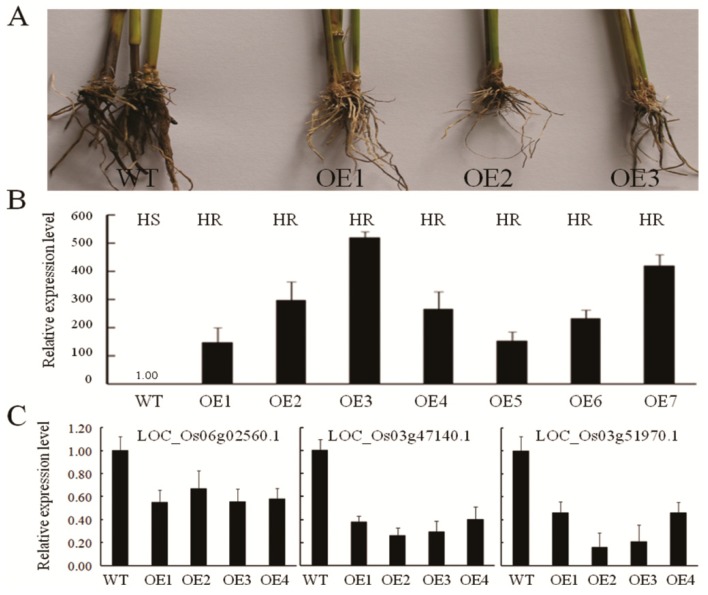
Overexpression of the osa-miR396f precursor in rice enhanced resistance to *D. zeae*. (**A**) Resistant phenotype to *D. zeae* in overexpressing transgenic plants; (**B**) The relative expression level of osa-miR396f precursor and resistant reactions in overexpressing rice. HS, highly susceptibility. HR, highly resistance; (**C**) The expression levels of three targets of osa-miR396f were inhibited in overexpressing rice. WT, wild-type rice Nipponbare. OE1-7, the osa-miR396f precursor- overexpressing rice plants.

To validate if osa-miR396f was involved in defense response to *D. zeae*, the precursor sequences of osa-miR396f were isolated from the resistant cultivar Nanjing 40 and were expressed under the control of the constitutive CMV35S promoter and transformed into susceptible japonica rice cultivar Nipponbare. Seven positive osa-miR396f precursor-overexpressing rice plants were inoculated with *D*. *zeae*. The transgenic rice plants with the osa-miR396f precursor showed increased resistance to *D*. *zeae* in comparison with that of Nipponbare (Figure 7A). Consistently, the transcripts of osa-miR396f precursor in all seven independent transgenic lines were higher than that in wild-type plants (Figure 7B). As predicted, transcripts of all three targets *OsGRFs* of osa-miR396f were significantly reduced in these transgenics revealed by qRT-PCR (Figure 7C). These results suggest that miRNA396f is involved in resisting *D*. *zeae* infection in rice.

## 3. Discussion 

Recently, rice bacterial foot rot disease caused by *D. zeae* has the potential to become one of the most important bacterial diseases, resulting in the loss of yield and grain quality in China, in the southeast of Asia and worldwide. In the present study we identified and characterized a number of miRNAs-target pairs involved in defense responses in rice by miRNA, degradome sequencing and qRT-PCR. 

The fast evolved high-throughput sequencing technology allows rapid identification of miRNAs and targets in plants that are involved in plant immunity [3,31]. Accumulated studies suggest that miRNAs played an important role for regulating the biological process and stress responses in plants [32,33]. It is known that plant miRNAs expressed differently during the infection process of pathogens [7]. In the present study, we found 652 miRNAs including 79 significantly differentially expressed miRNAs at different time points post inoculation in an incompatible interaction (Figure 2A; Table S3). For instance, two miRNAs miR827n-5p and miR164 we identified were also identified in a resistant rice variety to *M. grisea*, as negative and positive regulator respectively [21]. Moreover, the expression levels of seven miRNAs, miR156a, 159b, 166e-3p, 394, 396c-3p, 812 and 827 were decreased in rice in resistance response to *Xoo* [20]. We showed a similar responsive mode of these miRNAs to fungal and bacterial pathogens infection demonstrating that miRNAs were indeed involved in resistance response to *D*. *zeae* in rice. We also validated the expression profiles of nine miRNAs (miR2118, 393, 396, 166, 171, 156, 535, 159 and 5072) related to defense responses by qRT-PCR (Figure 5). The relevant targets of these nine miRNAs were also identified and verified by degradome sequencing and qRT-PCR (Table S6; Figure 6). These 9 miRNAs were previously shown to respond to the pathogens infection in an incompatible interaction, probably resulting in the activation of multiple defense responses [34,35,36,37,38]. Our data suggest that these nine differentially expressed miRNAs may be involved in regulating the resistance to *D*. *zeae* in rice.

In the present study, we showed that overexpression of osa-miR396f precursor in rice enhanced resistance to *D. zeae*, correlated with significant reduction of transcripts of three predicted targets *OsGRFs* (Figure 7). Therefore, miR396 may modulate resistance to *D*. *zeae* by cleaving the transcripts of its targets *OsGRFs*. Rice miR396 family has eight members, osa-miR396a, 396b, 396c, 396d, 396e, 396f, 396g and 396h in the miRNA database (miRBase, http://www.mirbase.org/, 27 March 2018). Rice miR396-*GRFs* pair was previously shown to regulate plant growth and development by positively activating plant horm*on*es indole-3-acetic acid (IAA), gibberellin (GA) and brassinolide biosynthesis pathways in rice [39,40,41,42]. The miR396 family members, osa-miR396e-3p and osa-miR396d/e-5p were induced by blast pathogen in a susceptible rice variety, and the expression level of osa-miR396c-5p was increased in a resistant rice variety [21]. Some members of miR396 were previously predicted to be a negative factor for the infection process of southern rice black-streaked dwarf virus (SRBSDV) in rice [38]. In contrast, overexpression of miR396a or miR396b significantly compromised the susceptibility to cyst Nematode in *Arabidopsis* [43]. The *Growth*-*Regulating Factors* (*GRFs*) were also the targets of miR396 family members and positively regulated the number and size of the rice grain [44]. The blocking of *OsGRFs* by miR396 generated a larger grain size, auxiliary branches and spikelets then enhanced grain yield through auxin in rice [41,45,46]. We speculate that miR396-*OsGRFs* pair may play a role for regulating the balance between yield and disease resistance via the auxin signaling pathway. However, the biological functions of miR396 on defense responses and rice grain development still need to be explored.

Plant miRNAs were predicted to be involved in the biological process and stress responses by inhibiting the transcripts of relevant targets [1,13,33]. In the present study, 132 miRNA responsive to the infection of *D. zeae* in an incompatible interaction were identified, and the predicted targets of 29 differentially expressed miRNAs were identified by degradome sequencing (Table S6). These identified targets might modulate rice resistance response to *D. zeae.* Most of the targets we identified were predicted to be involved in multiple defense response signal pathways such as *OsGRFs*, Leucine-rich repeat protein kinase, MYB domain protein, and auxin efflux carrier family protein (Table 2 and Table 3; Table S6). The genes, *AtGRF1* and *AtGRF3* were shown to be involved in multiple resistance-related signal pathways related with cell-wall modification, cytokinin biosynthesis and the accumulation of secondary metabolites in *Arabidopsis* [43,47]. Overexpression of miR396a or miR396b and miR396 targets *grf1*/*grf2*/*grf3* triple mutants previously significantly compromised the susceptibility to cyst nematode in *Arabidopsis* [43]. The miR396-*GRF6* interaction network was predicted to be involved in the development of inflorescence architecture by regulating the auxin biosynthesis and signaling pathways [41]. Moreover, the auxin-responsive *GH3* family gene LOC_Os07g40290.1 as the target of osa-miR172d-5p_R-2 showed more cleavages in a resistant rice variety after inoculation with *D. zeae*, compared with that without inoculation (Table 2). Additionally, auxin as a negative regulator was shown to contribute to resistance to bacteria pathogen *Xoo*, which resulted in the change of the cell wall structure [45,46]. The *Mybs1* gene encoding one MYB transcription factor was involved in a broad-spectrum resistance against *M*. *oryzae* in rice by increasing the accumulation of H_2_O_2_ through inhibition of the expression level of *bsr*-*d1* [48]. Taken together, these findings suggest that miRNA and targets are involved in multiple defense responses, including auxin and active oxygen signal pathways.

In summary, knowledge of miRNAs and targets opens a new avenue to understand the complexity of plant immunity. In the present study, a group of differentially expressed miRNAs and targets in an incompatible interaction at early interphases of host-pathogen interaction was identified by miRNA and degradome sequencing and qRT-PCR. These miRNAs and targets were predicted to be involved in multiple defense signal and diverse cellular pathways in rice. Through a transgenic approach, we demonstrated that one of these miRNAs, osa-miR396f enhanced the defense responses against *D*. *zeae* by inhibiting the transcripts of the three growth related transcription factor genes, *OsGRFs*. These results not only pave the road for controlling the newly emerging damaging foot rot disease but also promise a better understanding of plant innate immunity.

## 4. Materials and Methods

### 4.1. Plant Materials and Measurement of Resistance Reactions to D. zeae

The foot rot disease resistant *Japonica* rice variety Nanjing 40 was used to analyze the candidate miRNAs involved in disease resistance and the relevant targets by high-through sequencing technologies. The individual positive rice lines overexpressing the osa-miR396f precursor were chosen for analyzing disease reactions to *D. zeae*. All the plants used in this study were grown in a greenhouse in Jiangsu Academy of Agricultural Sciences, Nanjing, Jiangsu Province, China. Disease reactions to *D. zeae* were measured by the basal stem and root inoculation methods, respectively [30,49]. Disease reaction was classified as five groups by measuring the percentage of diseased area at 7–10 days after inoculation. No visible disease symptom in the stem and foot indicates immunity. The disease index less than or equal to five indicates highly resistance (HR). The disease index 5.1 to 12.4 indicates moderate resistance (MR). The disease index, 12.5 to 19.9 indicates moderate susceptibility (MS). The disease index greater than or equal to 20 indicates highly susceptibility (HS) [30,49]. 

### 4.2. Construction of Small RNA Library, Sequencing and Data Analysis

Total RNA was extracted from four mixed rice roots at 0 (K0), 6 (K1), 12 (K2) and 48 (K3) hpi with *D. zeae* using Trizol reagent (Invitrogen, Shanghai, China) following the manufacturer’s procedure respectively. The rice roots sampled with two biological repeats were combined as K0, K1, K2 and K3 for total RNA extraction, small RNA library construction and sequencing. Approximately 1 mg of total RNAs was used to prepare the miRNA library according to the protocol of TruSeq Small RNA Sample Prep Kits (Illumina, San Diego, CA, USA). The libraries were sequenced with the single-end sequencing (36 bp) on an Illumina Hiseq2500 at the LC-Bio Co., Ltd (Hangzhou, China) following the vendor’s recommended protocol.

The raw reads were analyzed with the Illumina pipeline filter (Solexa 0.3), and the dataset was further processed with an in-house program, ACGT101-miR (LC Sciences, Houston, TX, USA) to remove adapter dimers, junk, low complexity, common RNA families (rRNA, tRNA, snRNA, snoRNA) and repeats. Subsequently, unique sequences with length in 18–25 nucleotides were mapped to specific species precursors in the miRBase database (http://www.mirbase.org/, 27 March 2018) using the BLAST algorithm to identify known miRNAs and novel 3p and 5p-derived miRNAs. The unique sequences mapping to specific species mature miRNAs in hairpin arms were identified as known miRNAs; the unique sequences mapping to the other arm of known specific species precursor hairpin opposite to the annotated mature miRNA-containing arm were considered to be novel 5p or 3p-derived miRNA candidates [50,51]. 

### 4.3. Identification and Function Analysis of Targets of miRNAs

The total RNA extracted from control T0h (0 hpi) and infected T6–48h (mixtures of 6, 12, 48 hpi) rice sample respectively were used for degradome sequencing on Illumina Hiseq 2500 at the LC-Bio Co., Ltd. (Hangzhou, China). The degradome reads were mapped to the rice transcriptome data. Publicly available software packages, CleaveL and 3.0 pipeline was used to predict and identify the potentially cleaved targets [50,52]. Then, the computational target prediction algorithms (Target Finder) were used to identify miRNA binding sites [53]. All of the predicted target genes were analyzed with NCBI BLASTX algorithm. Finally, the GO terms of these differentially expressed miRNA targets were also annotated by AgriGO program. The candidate miRNAs-related to defense responses and their targets with differential expression based on normalized deep-sequencing counts were analyzed by the Fisher exact test, Chi-squared test, Student’s *t* test, and ANOVA based on the experiments design [54]. The level of significance threshold was set at 0.01 and 0.05 for each test.

### 4.4. Validation of Differentially Expressed miRNAs and Targets by qRT-PCR

To validate miRNAs and their target expressions determined by high-through sequencing technology, the root fragments at different time points after inoculation were used to extract total RNA using the Trizol reagent (TaKaRa, Dalian, China). The qRT-PCR analysis was performed with the Applied Biosystems 7500 Real Time PCR System and SYBR *Premix Ex Taq*^TM^ (TaKaRa, Dalian, China) according to the manufacturer’s instructions. The rice gene *EF1-a* and *U6* was used as the internal reference gene to standardize RNA quantity for evaluating relative expression levels. For qRT-PCR assays, three independent biological samples were carried out with three technical replicates with a gene-specific primer (Table S7).

### 4.5. Vector Construction and Rice Transformation

The 176nt precursor sequence of osa-miR396f was isolated from Nanjing 40 by PCR amplification, and inserted into the pCAMBIA1301 binary vector driving by the constitutive CMV35s promoter for overexpression. The T-DNA recombinant plasmids were transformed into calli derived from the mature Nipponbare embryos by the *Agrobacterium* mediated transformation method [46].

## Figures and Tables

**Table 1:** Overview of sequencing reads from raw data to cleaned sequences in four libraries.

Sequence Type	K0		K1		K2		K3	
	Total Counts	Unique Counts	Total Counts	Unique Counts	Total Counts	Unique Counts	Total Counts	Unique Counts
Raw reads	6,166,878	2,434,380	8,949,370	2,363,366	7,312,871	2,301,987	5,565,701	1,511,820
3ADT&length filter	1,909,403	1,024,342	2,458,847	982,458	2,232,137	922,159	2,525,190	803,851
Junk reads	18,778	13,765	21,772	14,658	25,064	16,132	12,782	9,015
Non-coding RNA	559,796	169,225	600,364	129,071	625,544	132,521	359,131	91,856
Rfam	888,552	201,522	1,146,506	162,399	1,071,523	164,209	637,768	115,524
mRNA	1,279,886	170,257	2,684,763	234,424	1,778,770	204,532	1,146,100	122,153
Repeats	13,479	3,241	19,257	2,241	14,299	2,895	9,875	1,869
rRNA	641,748	127,477	857,965	102,851	749,376	98,155	491,401	73,325
tRNA	176,213	57,003	173,576	39,418	228,124	45,153	91,910	27,900
snoRNA	12,162	4,408	33,516	6,862	21,275	5,829	10,543	3,854
snRNA	9,586	2,845	24,758	3,638	14,329	3,441	7,713	2,389
Other Rfam RNA	48,843	9,789	56,691	9,630	58,419	11,631	36,201	8,056
Clean reads	2,385,536	1,053,550	3,164,367	1,000,514	2,637,057	1,023,748	1,512,623	483,076

**Table 2:** The identified targets involved in rice defense responses by degradome sequencing.

No.	Targets	Target Annotation	UP/Down	Cleavage Site
1	LOC_Os08g40900.1	Auxin response factor 6	up	3466
2	LOC_Os07g40290.1	Auxin-responsive GH3 family protein	up	2091
3	LOC_Os06g39590.1	AUX/IAA transcriptional regulator	up	502
4	LOC_Os03g51970.1	Growth-regulating factor 1	up	430
5	LOC_Os11g35030.1	Growth-regulating factor 2	up	880
6	LOC_Os02g53690.1	Growth-regulating factor 5	up	581
7	LOC_Os04g04330.1	Leucine-rich repeat kinase	down	3509
8	LOC_Os10g33940.1	Auxin response factor 16	down	2332
9	LOC_Os12g10740.1	Leucine-rich repeat protein kinase	down	1219
10	LOC_Os11g38440.1	NB-ARC disease resistance protein	down	3382
11	LOC_Os04g59430.1	Auxin response factor 16	down	1345
12	LOC_Os04g38720.1	NAC domain containing protein 80	down	811
13	LOC_Os08g10080.1	NAC domain containing protein 1	down	783
14	LOC_Os12g40900.1	AUX/IAA transcriptional regulator	down	536
15	LOC_Os07g42510.1	Ethylene-responsive binding protein	down	721
16	LOC_Os03g08050.1	GTP binding Elongation factor Tu protein	down	969
17	LOC_Os03g08020.1	GTP binding Elongation factor Tu protein	down	969
18	LOC_Os03g08010.1	GTP binding Elongation factor Tu protein	down	969
19	LOC_Os06g01620.1	GRAS family transcription factor	down	464
20	LOC_Os02g44360.1	GRAS family transcription factor	down	1362
21	LOC_Os02g09060.1	Zinc finger family protein	down	401
22	LOC_Os08g41290.1	Auxin-responsive family protein	down	178
23	LOC_Os04g51560.1	WRKY DNA-binding protein 11	down	623
24	LOC_Os10g01100.1	Lectin protein kinase family protein	down	2638
25	LOC_Os02g50960.1	Auxin efflux carrier family protein	down	2109
26	LOC_Os12g37760.1	NB-ARC disease resistance protein	down	3101
27	LOC_Os07g33480.1	Cytochrome P450 family 716	down	1606
28	LOC_Os02g48080.1	S-locus lectin protein kinase	down	1135
29	LOC_Os02g11980.1	Leucine-rich repeat protein kinase	down	3002
30	LOC_Os12g43640.1	Leucine-rich receptor-like protein kinase	down	3203
31	LOC_Os01g11340.1	Cytochrome P450, family 710	down	1694
32	LOC_Os02g14120.1	Leucine-rich repeat protein kinase	down	2050
33	LOC_Os12g01510.1	Leucine-rich repeat protein kinase	down	2123
34	LOC_Os12g37980.1	Leucine-rich repeat transmembrane kinase	down	2614

**Table 3:** The miRNA-target pairs in multi-defense responses detected by miRNA and degradome sequencing.

No.	miR_name	Targets	Target Annotation	T0 h	T6–48 h	Up/Down	Cleavage Site
1	Osa-miR2118e-	LOC_Os03g06680.1	Protein of unknown function 506	91.48	0	down	1608
	p5_1ss13TA	LOC_Os02g50960.1	Auxin efflux carrier protein	91.48	0	down	2109
2	Osa-miR393a	LOC_Os03g36080.1	–	1829.51	0	down	741
		LOC_Os05g05800.1	F-box/RNI-like protein	182.95	460.85	up	1708
		LOC_Os04g32460.1	Auxin signaling F-box 2	1234.92	172.82	down	2235
3	Osa-miR396f	LOC_Os02g53690.1	Growth-regulating factor 5	0	460.85	up	581
		LOC_Os03g51970.1	Growth-regulating factor 1	0	115.21	up	430
		LOC_Os11g35030.1	Growth-regulating factor 2	0	115.21	up	880
4	Osa-miR166m_R-1	LOC_Os12g41860.1	Leucine zipper family protein	640.33	76.81	down	888
		LOC_Os03g43930.1	Leucine zipper family protein	548.85	76.81	down	966
		LOC_Os10g33960.1	Leucine zipper family protein	922.38	249.63	down	935
		LOC_Os03g01890.1	Leucine zipper family protein	899.51	249.63	down	1100
5	Osa-miR171b	LOC_Os02g44370.1	GRAS family transcription factor	457.38	0	down	1537
		LOC_Os02g44360.1	GRAS family transcription factor	274.43	0	down	1362
		LOC_Os10g40390.1	GRAS family transcription factor	182.95	0	down	179
		LOC_Os06g01620.1	GRAS family transcription factor	1189.18	460.85	down	467
		LOC_Os04g46860.1	GRAS family transcription factor	2378.36	691.27	down	1337
6	Osa-miR156a	LOC_Os08g41940.1	SBP transcription factor	457.38	0	down	1064
		LOC_Os01g69830.1	SBP transcription factor	182.95	115.21	down	1163
		LOC_Os02g04680.1	SBP transcription factor	457.38	57.61	down	1975
		LOC_Os11g30370.1	SBP transcription factor	91.48	0	down	1101
		LOC_Os09g31438.1	SBP transcription factor	91.48	0	down	819
		LOC_Os02g07780.1	SBP transcription factor	45.74	0	down	997
		LOC_Os06g49010.1	SBP transcription factor	45.74	0	down	1696
		LOC_Os06g45310.1	SBP transcription factor	274.43	115.21	down	864
7	Osa-miR535-5p	LOC_Os06g45310.1	Squamosa promoter-like 11	91.48	0	down	863
8	Osa-miR159a_1R-3	LOC_Os01g59660.1	Myb domain protein 33	411.64	288.03	down	135
		LOC_Os10g05230.1	RING/U-box superfamily protein	274.43	0	down	1271
		LOC_Os12g10740.1	Leucine-rich repeat protein kinase	182.95	0	down	346
		LOC_Os06g40330.1	Myb domain protein 65	182.95	0	down	1219
		LOC_Os01g47530.1	MAP kinase 20	68.61	28.80	down	2333
9	Osa-miR5072 _L-4	LOC_Os05g07050.1	Pre-mRNA processing splicing factor	45.74	0	down	5043

## Supplementary Materials

Supplementary materials can be found at http://www.mdpi.com/1422-0067/20/1/222/s1.
